# Psychotropic use patterns: Are there differences between men and women?

**DOI:** 10.1371/journal.pone.0207921

**Published:** 2018-11-26

**Authors:** Camila Stéfani Estancial Fernandes, Renata Cruz Soares de Azevedo, Moisés Goldbaum, Marilisa Berti de Azevedo Barros

**Affiliations:** 1 Department of Public Health, School of Medical Sciences, State University of Campinas, Campinas, Sao Paulo, Brazil; 2 Department of Medical Psychology and Psychiatry, School of Medical Sciences, State University of Campinas, Campinas, Sao Paulo, Brazil; 3 Department of Preventive Medicine, Medical School, University of Sao Paulo, Sao Paulo, Brazil; Texas Technical University Health Sciences Center, UNITED STATES

## Abstract

This study analyzed differences between men and women regarding the use of psychotropic drugs and associated factors in a population of adults and seniors in the city of Campinas, Brazil. A population-based, cross-sectional study was conducted using data from the ISACamp 2014/2015 health survey in the city of Campinas. The sample was composed of 1999 individuals aged 20 years or older. For each sex, prevalence rates and prevalence ratios were estimated for the use of psychotropic drugs according to demographic characteristics, socioeconomic characteristics, health problems, degree of limitation and type of emotional/mental problem. The most used classes of medications were also determined. The prevalence of the use of psychotropic drugs was 11.7% (7.3% among men and 15.8% among women). The most common therapeutic classes were antidepressants (38.2%) and benzodiazepines (24.0%). The frequency of antidepressant use was higher among women (44.3%) than men (25.5%). Regarding associated factors, reports of emotional/mental problems were associated with the greater use of this type of drug in both sexes. Among the men, white skin color, a lack of an occupational activity, a greater number of complaints of health problems and the occurrence of insomnia were associated with the use psychotropic drugs. Among the women, a significant increase in the use of these drugs was found with the increase in age and higher prevalence rates were found among those with a higher level of schooling, those with a greater number of diagnosed chronic diseases and those with a common mental disorder. The present results confirm the greater use of psychotropic agents, especially antidepressants, in the female sex and reveal that the pattern of associated factors differs between sexes. It is therefore necessary to understand the peculiarities of each sex that exert an influence on the perception of health problems and the desire to seek care, which, in turn, affect the use of psychotropic agents.

## Introduction

The World Health Organization estimates that at least 10% of the world’s population is affected by mental disorders, the most common of which are anxiety and depression, which account for 30% of the non-fatal disease burden worldwide and 10% of the overall disease burden [1]. In Brazil, recent data from the National Health Survey indicate that 9.7% of adults had some degree of depression in the two weeks prior to being interviewed [2]. Differences are generally found in the prevalence of mental disorders between men and women. Mood disorders and anxiety are more prevalent among women and substance use disorders are more common among men [3,4].

Considering the prevalence and relevance of mental disorders, psychotropic medications constitute an important tool in the treatment and control of diverse psychopathological conditions [5]. However, there is evidence of the high and increasing prevalence of the consumption of these medications [6–8], raising questions with regard to possible excessive use and a lack of rational use. On the other hand, a large number of individuals with mental disorders remain without medicinal treatment in particular segments of the population. The gap between the demand and use of these medications is important, as this difference can be as high as 80 to 85%, depending on the location of the study [9,10].

Studies conducted in Brazil and other countries report prevalence rates of the use of psychotropic drugs ranging from 9.9 [7] to 15.6% [11] for reported consumption in the previous 15 days and from 7.1 [12] to 14.9% [13] in a 12-month recall period. These studies generally point out factors associated with the use of such drugs, such as an advanced age [7,10,12], the active seeking of health services [6,7,14], a poor self-assessment of one’s health [6,14] and greater use among women in comparison to men [3,10,11]. The magnitude of the difference in the use of these drugs between the sexes varies across countries and also depends on the recall period investigated. In a study conducted in 2015 in ten European countries, the annual prevalence of the use of psychotropic drugs was 15.1% among women and 8.0% among men [3]. In the United States of America (USA), the National Health and Nutrition Examination Survey (NHANES, 2012) found greater use of anxiolytic agents among women than men in the 30 days prior to the survey (6.8% versus 5.3%) [8]. Studies conducted in Brazil have also found a greater use of psychotropic drugs among women compared to men [9,10,12,15,16], with prevalence ratios ranging from 1.5 [16] to 2.8 [9], depending on the location and recall period studied.

Investigations of the use of psychotropic drugs employing data from population-based studies are justified by the high and increasing prevalence of the consumption of these drugs in particular segments of society, especially anxiolytic agents and antidepressants [7,16]. Although the literature has documented greater use of these medications among women, few population-based studies have investigated differences between sexes regarding factors associated with the use of these medications.

Therefore, the aim of the present study was to analyze differences between men and women regarding the use of psychotropic drugs and factors associated with this use among adults and seniors in the city of Campinas, Brazil. A further aim was to identify the most used classes of medications.

## Methods

### Study design and target population

A population-based, cross-sectional study was conducted involving the analysis of data from a sample of 1999 residents of the city of Campinas, Brazil, aged 20 years or older. Campinas is a large city located in the state of São Paulo (southeastern Brazil) with a population estimated at 1,164,098 inhabitants in 2015, 98.3% of whom reside in urban areas. The Human Development Index was 0.805 in 2010 [17]. The data used in the present study were extracted from the Campinas Health Survey (hereafter denominated ISACamp) conducted in 2014/15. The aim of the survey was to analyze different dimensions of health, including health status, diseases, health behavior, the use of health services and the use of medications.

### Sampling and data collection

The sample was obtained using stratified probabilistic cluster sampling in two stages: census sector and residence. In the first stage, 14 census sectors were selected in each of the five administrative districts of the city, totaling 70 sectors. The sectors were selected with probability proportional to size given by the number of residences per sector and lists of homes in each sector were updated. In the second stage, residences were selected in each sector using a systematic lottery applied to the updated lists of homes.

Considering the aim of studying aspects related to three subpopulations in the city of Campinas (adolescents, adults and seniors), the following age groups constituted the domains of the study: 10 to 19 years, 20 to 59 years and 60 years or older. The number of individuals to compose the sample was obtained considering the estimate of a 50% proportion (corresponding to maximum variability), 95% confidence interval, 4 and 5% sampling error and a design effect of 2, resulting in 1000 adolescents, 1400 adults and 1000 seniors. To obtain the desired sample size, 3119, 1029 and 3157 residences were selected independently for interviews with adolescents, adults and seniors, respectively, considering non-response rates of 27%, 22% and 20% for the three age groups, respectively. At each home, all residents in the particular age group were interviewed. The decision not to perform intra-residence selection in the field was due to the fact that this type of design is similar in terms of precision and is less costly in comparison to performing the selection of an interviewee per residence [18]. Further details on the sampling process are available on the following webpage: https://www.fcm.unicamp.br/fcm/ccas-centro-colaborador-em-analise-de-situcao-de-saude/isacamp/2014.

Data were collected with the aid of a pre-coded questionnaire that contained predominantly closed-ended questions organized in 11 thematic blocks. Data collection was performed in interview format with the selected individuals by trained interviewers and the data were recorded on a portable computer (tablet).

### Variables analyzed

In the present study, data on individuals aged 20 years or older were used. The dependent variable was the use of psychotropic drugs in the 15 days prior to the survey and was dichotomized as none (0) and one or more psychotropic drugs (1), which was determined through the following question: *Have you taken any medication in the last 15 days*? If an affirmative response was given, the following question was posed: *How many and which medications did you take*?

To minimize the occurrence of errors when recording the data, the medications were identified (the majority of times) based on the label of the medication and/or medical prescription. The Dictionary of Pharmaceutical Specialties [19] was used to identify the active ingredient in each medication and the Anatomical Therapeutical Chemical Classification System (ATC) [20] was then used to code the medications based on the active ingredient (chemical substance). With the ATC, medications are classified on five levels. The first level regards the main anatomic group. The second level corresponds to the therapeutic subgroup. The third and fourth levels refer to pharmacological or therapeutic subgroups and the fifth level is the chemical substance. In the present study, the psychotropic drugs were identified based on the chemical substance (fifth level of the ATC) and grouped according to the pharmacological/therapeutic subgroup (third or fourth level of the ATC). Medications pertaining to the following ATC classifications were analyzed: antidepressants (N06A), benzodiazepines (N03AE, N05BA, N05CD and N05CF), antiepileptics (N03A) and other classes composed of opioid analgesics (N02A), anti-Parkinson’s drugs (N04A and N04B), antipsychotic agents, such as mood stabilizers (N05A), psychostimulants (N06B) and anti-dementia drugs (N06D).

The following sets of independent variables were selected for the analysis of factors associated with the use of psychotropic drugs:

- Demographic and socioeconomic characteristics: age group (20 to 39 years, 40 to 59 years or 60 years or older), schooling (0 to 4 years, 5 to 11 years or 12 or more years of study), skin color/ethnicity (white or non-white [black and brown]), paid occupational activity (yes or no), household income *per capita* (categorized based on the Brazilian monthly minimum wage [BMMW]: ≤ BMMW, one to three times the BMMW or > three times the BMMW) and having a private health insurance plan (yes or no).
- Health problems: Number of diagnosed chronic diseases on the following checklist: arterial hypertension, diabetes mellitus, cardiovascular disease (angina, myocardial infarction and cardiac arrhythmia), cancer, arthritis/rheumatism/arthrosis, osteoporosis, rhinitis/sinusitis, asthma/bronchitis/emphysema, tendonitis/repetitive strain injury/work-related musculoskeletal disorder, vascular problems (varicose veins and stroke), hypercholesterolemia and back disease/problem; reported number of health problems (complaints and symptoms), such as headache/migraine, back pain/problem, allergy, dizziness/vertigo, urinary problem (urinary tract infection/cystitis and urinary incontinence), insomnia and degree of limitation caused by insomnia (no limitation, some limitation or considerable limitation).
- Emotional health: Common mental disorders (CMDs) were evaluated using the Self-Reporting Questionnaire (SRQ 20), which is composed of 20 items with dichotomous responses. A cutoff point was 7 was used for both sexes [21] (0 to 6 points denote absence of CMD; 7 or more points denote presence of CMD). Severity was determined as follows: 0 to 6 points = absence of CMD, 7 to 10 points = moderate CMD and 11 or more points = severe CMD. The following were also evaluated: report of emotional/mental problem (yes or no); degree of limitation caused by this problem (no limitation, some limitation or considerable limitation) and type of reported emotional/mental problem (anxiety, depression or other).

### Data analysis

All analyses performed in the present study considered the weights stemming from the complex sampling design and non-response weights. For such, the survey *(svy)* model of Stata 14.0 (Stata Corp., College Station, USA) was used.

Pearson’s chi-squared test was used to determine associations between the independent variables and the use of psychotropic agents, with the level of significance set to 5%. Crude and adjusted prevalence ratios were estimated using Poisson regression, which is considered an adequate choice for analyzing data from cross-sectional studies with binary outcomes [22]. Variables with a p-value < 0.20 in the bivariate analysis were incorporated into the hierarchical model using the stepwise backward method and those with a p-value < 0.05 after the adjustments remained in the final model. Significant variables in one step remained in following steps even if losing their significance. The complete model included demographic and socioeconomic characteristics (first step), health problems (second step) and emotional health and insomnia (third step). The pharmacological classes of the psychotropic agents were also identified and the percentage frequencies of antidepressants, benzodiazepines, antiepileptics and other classes were determined.

### Ethical aspects

This study received approval from the Human Research Ethics Committee of Campinas State University (certificate number: 409.714 of September 30^th^, 2013). All interviewees signed a statement of informed consent.

## Results

Among the residences selected for interviews with adults and seniors, refusal and dropout rates were 7.4% and 4.4%, respectively. Among the adults and seniors identified in the residences selected for interviews, the refusal and dropout rates were 20.5% and 1.9%, respectively. Therefore, the data from 1999 individuals aged 20 years or older were analyzed. Men (mean age: 42.6 ± 0.8 years) accounted for 47.3% of the sample and women (mean age: (44.7 ± 0.7 years) accounted for 52.7%.

The prevalence of the use of at least one medication in the previous 15 days was 65.7% and was significantly higher among the women (75.7%) than the men (54.6%) (p = 0.0000). The prevalence of the use of psychotropic agents in the population of adults and seniors in the city of Campinas was 11.7%, with rates of 7.3% (CI: 5.5 to 9.6%) among the men and 15.8% (CI: 13.1 to 18.8%) among the women. After controlling for age, the prevalence ratio between sexes was 2.03 (CI: 1.45 to 2.85). Among those who took psychotropic agents, 68.5% used only one type, 21.1% used two types and 10.2% used three or more types simultaneously. Using data from the SEADE (State of São Paulo Data Analysis System) Foundation in 2015 [23], the present findings indicate that among the 849,672 inhabitants of the city of Campinas aged 20 years or older, 99,412 individuals made use of some type of psychoactive drug during the study period. For both sexes, higher prevalence rates of use were found among individuals aged 60 years or older, those with white skin and those who reported not performing a paid occupational activity during the study period. Among the women, having a private insurance plan was also positively associated with the use of psychotropic agents (Table 1).

**Table 1:** Prevalence of the use of psychotropic agents and prevalence ratios according to sex and demographic/socioeconomic characteristics. Campinas, Brazil, 2014–2015.

Variables	Male sex			Female sex		
	n	Prevalence %	PR (95%CI)	n	Prevalence %	PR (95%CI)
**Age (years)**		(0.0069)*χ^2^			(0.0000)*χ^2^	
20–39	258	4.3	1	291	9.9	1
40–59	206	9.6	2.24 (1.13–4.41)*	258	16.3	1.65 (1.00–2.70)
60 or older	387	11.5	2.68 (1.38–5.20)*	599	28.0	2.84 (1.86–4.34)*
Total	851	7.3		1148	15.8	
**Schooling (in years)**		(0.6985)			(0.0188)*	
0–4	282	8.7	1	458	18.7	1
5–11	412	7.3	0.84 (0.49–1.44)	495	12.2	0.65 (0.46–0.91)*
12 or more	157	6.5	0.74 (0.35–1.56)	195	20.0	1.07 (0.69–1.65)
**Skin color/ethnicity**		(0.0060)*			(0.0126)*	
Non-white	265	3.0	1	347	11.1	1
White	568	9.6	3.19 (1.33–7.65)*	773	17.7	1.60 (1.10–2.33)*
**Paid occupational activity**		(0.0001)*			(0.0207)*	
Yes	476	4.7	1	465	13.3	1
No	374	13.8	2.92 (1.71–4.99)*	683	19.1	1.43 (1.05–1.96)*
**Household income *per capita* (in BMMW)**		(0.1608)			(0.8367)	
≤ 1	265	5.2	1	422	14.7	1
> 1- ≤ 3	457	9.1	1.75 (0.96–3.16)	574	16.1	1.01 (0.68–1.76)
> 3	119	6.1	1.17 (0.44–3.07)	151	17.1	1.16 (0.69–1.94)
**Private health insurance plan**		(0.1832)			(0.0261)*	
No	508	6.1	1	603	13.1	1
Yes	342	8.9	1.45 (0.83–2.53)	545	18.5	1.40 (1.04–1.89)*

n: number of individuals in unweighted sample.

* statistically significant difference.

χ^2^ between parentheses: p-value from the chi-squared test.

PR (95%CI): Prevalence ratio (95% confidence interval).

In both sexes, the prevalence of use increased with the increase in the number of chronic diseases and health problems. The prevalence was higher among individuals with complaints of insomnia, emotional/mental problems and CMDs (Table 2).

**Table 2:** Prevalence of the use of psychotropic agents and prevalence ratios according to sex and health problems. Campinas, Brazil, 2014–2015.

Variables	Male sex			Female sex		
	n	Prevalence (%)	PR (95%CI)	n	Prevalence (%)	PR (95%CI)
**Number of chronic diseases**		(0.0396)*χ^2^			(0.0000)*χ^2^	
0	252	5.8	1	231	10.8	1
1–2	360	6.6	1.14 (0.64–2.10)	407	9.6	0.89 (0.51–1.56)
3–4	166	9.8	1.68 (0.71–3.98)	292	22.8	2.11 (1.19–3.74)*
5 or more	72	17.4	3.00 (1.38–6.53)*	215	33.9	3.14 (1.89–5.19)*
**Number of health problems**		(0.0015)*			(0.0314)*	
0	398	5.9	1	351	10.4	1
1	275	5.1	0.87 (0.43–1.76)	322	17.3	1.66 (1.07–2.57)*
2 or more	177	14.6	2.47 (1.30–4.68)*	472	18.1	1.73 (1.12–2.67)*
**Insomnia**		(0.0000)*			(0.0001)*	
No	689	4.4	1	800	11.8	1
Yes	161	21.3	4.82 (2.33–9.94)*	345	26.9	2.28 (1.51–3.43)*
**Emotional/mental problem**		(0.0000)*			(0.0000)*	
No	624	3.5	1	697	6.6	1
Yes	222	18.3	5.15 (3.01–8.80)*	448	29.4	4.44 (3.17–6.21)*
**Common mental disorder**		(0.0000)*			(0.0000)*	
No	748	5.2	1	862	11.7	1
Yes	74	22.8	4.36 (2.38–7.98)*	258	28.8	2.47 (1.70–3.58)*

n: number of individuals in unweighted sample.

* statistically significant difference.

χ^2^ between parentheses: p-value from the chi-squared test.

PR (95%CI): Prevalence ratio (95% confidence interval).

The results of the multivariate analysis revealed that reported emotional/mental problems were associated with the greater use of psychotropic agents in both sexes. Among the men, white skin color, a lack of an occupational activity, a greater number of complaints of health problems and the occurrence of insomnia were positively associated with the use psychotropic drugs. Among the women, a significant increase in the use of these drugs was found with the increase in age and higher prevalence rates were found among those with more than 11 years of schooling, those with a greater number of diagnosed chronic diseases and those with CMDs (Table 3).

**Table 3:** Multiple Poisson regression models of the use of psychotropic agents among men and women. Campinas, Brazil, 2014–2015.

Variables	First step^a^		Second step^b^		Third step^c^	
	PR (95%CI)	p	PR (95%CI)	p	PR (95%CI)	p
**Male sex**						
**Age (years)**						
20–39	1		1		1	
40–59	1.72 (0.87–3.39)	0.115	1.52 (0.77–2.98)	0.222	1.33 (0.73–2.40)	0.342
60 or older	1.36 (0,71–2.59)	0.352	1.20 (0.62–2.33)	0.587	1.27 (0.68–2.35)	0.448
**Skin color/ethnicity**						
Non-white	1		1		1	
White	2.94 (1.22–7.07)*	0.017*	2.91 (1.21–7.02)*	0.018*	2.45 (1.04–5.76)*	0.040*
**Paid occupational activity**						
Yes	1		1		1	
No	2.70 (1.54–4.74)*	0.001*	2.82 (1.61–4.94)*	0.000*	2.19 (1.28–3.74)*	0.005*
**Number of health problems**						
0			1		1	
1			0.89 (0.44–1.81)	0.738	0.80 (0.39–1.61)	0.519
2 or more			2.49 (1.36–4.58)*	0.004*	1.57 (0.83–2.96)	0.163
**Insomnia**						
No					1	
Yes					2.34 (1.05–5.24)*	0.038*
**Emotional/mental problem**						
No					1	
Yes					3.13 (1.68–5.83)*	0.001*
**Female sex**						
**Age (years)**						
20–39	1		1		1	
40–59	1.87 (1.14–3.06)*	0.013*	1.60 (0.96–2.67)	0.073	1.80 (1.08–2.98)*	0.023*
60 or older	3.81 (2.52–5.76)*	0.000*	2.72 (1.69–4.36)*	0.000*	3.49 (2.17–5.60)*	0.000*
**Schooling (in years)**						
0–4	1		1		1	
5–11	1.15 (0.82–1.61)	0.404	1.23 (0.87–1.74)	0.228	1.26 (0.85–1.86)	0.246
12 or more	2.17 (1.45–3.23)*	0.000*	2.20 (1.47–3.31)*	0.000*	2.51 (1.55–4.08)*	0.000*
**Number of chronic diseases**						
0			1		1	
1–2			0.78 (0.44–1.36)	0.371	0.62 (0.35–1.08)	0.091
3–4			1.59 (0.88–2.89)	0.124	1.05 (0.55–1.97)	0.888
5 or more			2.17 (1.25–3.77)*	0.007*	1.12 (0.61–2.07)	0.702
**Common mental disorder**						
No					1	
Yes					1.65 (1.10–2.47)*	0.015*
**Emotional/mental problem**						
No					1	
Yes					3.51 (2.65–4.64)*	0.000*

PR (95%CI): Prevalence ratio (95% confidence interval).

* statistically significant difference.

^a^: PR adjusted by demographic and socioeconomic variables.

^b^: PR adjusted for demographic and socioeconomic variables from first step and health variables.

^c^: PR adjusted by all variables in table.

Table 4 displays the prevalence of the use of psychotropic agents and prevalence ratios according to the occurrence of emotional/mental problems, CMDs and insomnia. In both sexes, greater use was associated with the degree of limitation caused by an emotional/mental problem and insomnia. Using the prevalence of use in cases of anxiety as reference, greater prevalence rates of use were found among women with a complaint of depression and men with a complaint of other mental/emotional problems, the highest frequencies of which were for schizophrenia (45.8%) and activity/attention disorders (32.9%). The occurrence of CMDs was associated the use of psychotropic agents in both sexes.

**Table 4:** Prevalence of the use of psychotropic agents and prevalence ratios according to sex and emotional/mental problem, common mental disorder and insomnia. Campinas, Brazil, 2014–2015.

Variables	Male sex			Female sex		
	n	Prevalence (%)	PR (95%CI)	n	Prevalence (%)	PR (95%CI)
**Limitation from emotional/mental problem**		(0.0000)*χ^2^			(0.0000)*χ^2^	
No problem	624	3.6	1	697	6.6	1
No limitation	110	9.2	2.58 (1.02–6.56)*	192	27.2	4.11 (2.78–6.09)*
Some limitation	79	19.4	5.45 (2.80–10.62)*	192	24.4	3.70 (2.48–5.50)*
Considerable limitation	33	43.3	12.18 (6.09–24.36)*	64	52.5	7.93 (5.24–12.00)*
**Type of emotional/mental problem**		(0.0000)*			(0.0000)*	
Anxiety	162	13.3	1	295	23.5	1
Depression	49	26.2	1.97 (0.99–3.89)	141	47.4	2.02 (1.48–2.75)*
Other problems	11	69.6	5.23 (2.64–10.36)*	12	43.4	1.84 (0.78–4.35)
**Common mental disorder (CMD)**		(0.0000)*			(0.0000)*	
No	748	5.2	1	862	11.7	1
Moderate CMD	56	22.6	4.32 (2.16–8.64)*	148	22.5	1.93 (1.17–3.17)*
Severe CMD	18	23.8	4.55 (1.73–11.91)*	110	37.2	3.19 (2.16–4.71)*
**Limitation from insomnia**		(0.0000)*			(0.0000)*	
Without insomnia	689	4.4	1	800	11.8	1
No limitation	65	9.9	2.24 (0.77–6.51)	137	20.2	1.72 (0.92–3.19)
Some limitation	62	25.9	5.87 (2.76–12.49)*	153	22.5	1.91 (1.12–3.24)*
Considerable limitation	34	37.3	8.47 (3.82–18.75)*	55	53.1	4.50 (2.99–6.78)*

n: number of individuals in unweighted sample.

* statistically significant difference.

χ^2^ between parentheses: p-value from the chi-squared test.

PR (95%CI): Prevalence ratio (95% confidence interval).

Table 5 displays the percentages of the classes of psychotropic agents, the most frequent of which were antidepressants (38.2%), with a significantly higher proportion among women (44.3%) in comparison to men (25.5%) (p = 0.0106). The antidepressants most used in the population of Campinas were selective serotonin reuptake inhibitors (28.8%), especially fluoxetine and sertraline, followed by non-selective monoamine reuptake inhibitors (tricyclic antidepressants) (6.2%), the most consumed of which was amitriptyline. Other antidepressants accounted for 3.2%. Benzodiazepines accounted for 24.0% of the psychotropic agents and the proportion between men and women was similar (24.1 and 24.0%, respectively). The most frequently used benzodiazepines were clonazepam and diazepam. Antiepileptics accounted for 15.4% of the psychotropic drugs, the most frequent of which was carbamazepine. Other psychotropic agents included opioid analgesics, anti-Parkinson’s, anti-psychotic and anti-dementia medications and psychostimulants. The frequency of these medications ranged from 1.7% (psychostimulants) to 7.3% (anti-psychotic medications). The most frequent anti-psychotic medications were quetiapine and haloperidol.

**Table 5:** Classes of psychotropic agents used (in %) according to sex; Campinas, Brazil, 2014–2015.

Classes of psychotropic agents according to ATC classification system	Total		Male sex	Female sex	p-value χ^2^
	n	%^a^	%^a^	%^a^	
**Antidepressants**	154	38.2	25.5	44.3	0.0106*
SSRIs^1^^#^	111	28.8	20.2	33.0	0.0756
Tricyclic^2^	25	6.2	3.90	7.3	0.3195
Others^3^	18	3.2	1.40	4.0	0.1556
**Benzodiazepines**	115	24.0	24.1	24.0	0.9930
Clonazepam	60	13.0	15.3	11.8	0.4095
Diazepam	29	6.0	6.8	5.7	0.7106
Others^4^	26	5.0	2.0	6.5	0.0580
**Anti-epileptics**	56	15.4	21.2	12.6	0.1302
Carbamazepine	20	5.2	5.8	5.0	0.8054
Phenytoin	9	1.8	3.5	0.9	0.0610
Others^5^	27	8.4	11.9	6.7	0.2377
**Other psychotropic agents**^6^	109	22.4	29.3	19.1	0.2743
Antipsychotics	30	7.3	13.1	4.5	0.0707
Anti-Parkinson’s	23	4.3	6.2	3.6	0.5763
Others	56	10.7	9.9	11.1	0.6754

n: number of medications.

^a^ Percentages weighted for sample design.

χ^2^ p-value from chi-squared test.

* statistically significant difference.

^1^ fluoxetine, citalopram, paroxetine, sertraline, escitalopram.

^2^ imipramine, clomipramine, amitriptyline, nortriptyline.

^3^ trazodone, mirtazapine, bupropion, venlafaxine, duloxetine, desvenlafaxine.

^4^ lorazepam, bromazepam, clobazam, alprazolam, cloxazolam, flunitrazepam, zolpidem.

^5^ phenobarbital, primidone, oxcarbazepine, valproic acid, lamotrigine, topiramate, gabapentin, pregabalin.

^6^ Opioid analgesics (morphine, codeine, tramadol); Anti-Parkinson’s (biperiden, levodopa, amantadine, pramipexole, selegiline, rasagiline, entacapone); Antipsychotics (levomepromazine, haloperidol, olanzapine, quetiapine, lithium, risperidone, aripiprazol); Anxiolytics (buspirone); Psychostimulants: (methylphenidate, piracetam); Anti-dementia (rivastigmine, galantamine, memantine).

^#^ selective serotonin reuptake inhibitors.

## Discussion

The findings of the present study demonstrate differences between men and women regarding factors associated with the use of psychotropic drugs. As expected, reports of an emotional and/or mental problem were associated with greater use in both sexes. Among men, white skin color, a lack of an occupational activity, a greater number of complaints of health problems and the occurrence of insomnia were positively associated with the use psychotropic drugs. Among women, a significant increase in the use of these drugs was found with the increase in age and higher prevalence rates were found among those with a higher level of schooling, those with a greater number of diagnosed chronic diseases and those with CMDs. Antidepressants and benzodiazepines were the most frequent therapeutic classes and the use of antidepressants was significantly higher among the women.

The prevalence of the use of psychotropic drugs in the present study was 11.7%. This is higher than the rate reported in a study conducted in the city of Pelotas in southern Brazil (9.9%) [7], which considered the same recall period and analyzed individuals aged 15 years or older. The prevalence was also higher than that reported in another study conducted in the city of Campinas using data from the 2008/2009 ISACamp health survey, in which a rate of 6.8% was found among adults and seniors in the three days prior to the survey [16], as well as two studies conducted in the city of Sao Paulo involving individuals aged 15 and 18 years or older, with rates of 5.5% in the previous 30 days and 7.1% in the previous 12 months [10,12]. In contrast, studies conducted in other countries report higher prevalence rates of the use of psychotropic drugs. A study conducted in Spain with individuals aged 25 years or older reports a prevalence rate of 15.6% in the previous 15 days [11]. A study conducted in Northern Ireland involving individuals aged 18 years or older found a prevalence rate of 14.9% in the previous 12 months [13]. The differences across studies may be related to differences in the recall period, age groups and data collection instruments as well as differences in the prevalence of emotional/mental problems, access to healthcare services and the prescribing habits of physicians.

The greater consumption of psychotropic drugs among women is in agreement with data described in previous studies [10,11,13,16]. Researchers have attributed this greater consumption to more frequent complaints of mental disorders, such as anxiety and depression, among women [24] as well as the fact that women are generally more attentive to signs and symptoms and seek medical care more often than men [25,26], which increases the probability of receiving prescriptions for psychotropic drugs. Gender may also affect the perceptions of physicians regarding the need for psychotropic drugs [27]. Qualitative studies report that physicians in primary care address the subjective symptoms of anxiety and depression differently depending on the patient’s sex. Beliefs regarding the greater frailty of the female sex lead to an unequal evaluation of the same health problems. Thus, physicians tend to diagnose emotional/mental problems more in women and prescribe them anxiolytic agents and antidepressants more [27,28].

The increase in age was associated with an increase in the use of psychotropic drugs only among the women in the present study, whereas the adjustment for occupational activity made the association between age and psychotropic drugs non-significant among the men. Previous studies evaluating the general population without analyzing the sexes separately have also found a positive association between the increase in age and the use of psychotropic drugs [9,10,12,13,16]. The high prevalence of mental disorders among women with the increase in age may be the result of interactions between biological changes and events in one’s psychosocial life [29]. Changes in estrogen levels during menopause and factors related to the redefinition of roles, such as the loss of fertility and the “empty nest” syndrome, are believed to be associated with the greater prevalence of mental disorders in this stage of life [29]. Moreover, the occurrence of comorbidities due to ageing [30] and the greater frequency of widowhood due to fact that women live longer than men [31] may contribute to an increase in depressive and anxious episodes, which could lead to a greater frequency of seeking medical care and consequently more opportunities for diagnosis and treatment.

A higher level of schooling was also associated with the use of psychotropic drugs only among the women. Previous studies report a positive association between a higher level of schooling and the use of these drugs in the general population [10,32], but not only among women. In the present investigation, the effect of schooling on access to medications may indicate that women with a higher level of education have more knowledge regarding health and healthcare services, perceive health needs more and have greater autonomy when seeking medical care and treatment [33].

Among the men, the use of psychotropic drugs was more frequent among whites compared to non-whites. This is a unique finding in the literature. Studies conducted in Brazil [16] and the USA [34,35] also report significantly lower use among non-whites, but in the general population and not only among men. The disparity in the consumption of psychotropic drugs among different racial groups has been attributed to injustices regarding access to health care and different cultural patterns, which may result in attitudes and thoughts of stigmatization against mental disease and a reluctance to seek treatment on the part of minority ethnic groups [36,37].

Men without a paid occupational activity used significantly more psychotropic drugs than those with paid work. This association was not found among the women. A study conducted in the city of Pelotas, Brazil, involving adults and seniors found a greater use of antidepressants among individuals who were not exercising any labor activity [38]. A study involving a nationally representative sample in Spain reports a similar finding [11]. However, these associations were found in the general population and not only among men. Participation in the job market, especially for men, means social participation [39] and validation as an active citizen [30] as well as ensuring better living conditions, health and emotional stability, whereas unemployment leads to a loss of purchasing power as well as economic insecurity [40]. Moreover, due to the fact that society traditionally attributes the role of worker/provider to men, they may harbor a notion of impotence when facing unemployment or retirement, which can exert a negative influence on mental health [41].

Having two or more health complaints was associated with a greater use of psychotropic drugs among the men in the present study. This finding may be related to the fact that men generally only seek healthcare services when a health problem is perceived and considered limiting [42], whereas women seek healthcare services more for routine examinations and prevention [43]. Among the women, having a greater number of chronic diseases was associated with the use of psychotropic drugs, which is in agreement with data described in previous studies [9,44]. Chronic conditions are generally more frequent among women [43,45,46] and can lead to functional disability, which has been correlated to the emergence of psychiatric symptoms [47]. As a result, women to seek healthcare services more, which increases the probability of receiving prescriptions for psychotropic agents [7].

Some factors associated with the use of psychotropic drugs differed between sexes. Among the women, CMDs were strongly associated with use, which is in agreement with data described in previous studies [25,44,48]. Among the men, adjustments for reported insomnia and emotional/mental problems made the association with CMDs non-significant. This demonstrates that a report of insomnia is a much stronger predictor of the use of psychotropic drugs than CMDs among men. The association with insomnia among men has not previously been reported in Brazilian studies, but has been found in a longitudinal study conducted in Finland [49]. It is likely that the acute consequences of insomnia, such as irritability, reduced performance, altered concentration and fatigue [50], exert a greater impact on the search for medical care among men.

As expected, reports of emotional/mental problems were strongly associated with the use of psychotropic drugs in both sexes. Use was more frequent among women with depression and men who reported other mental/emotional problems, especially schizophrenia and activity/attention disorders. Men often have greater difficulty expressing emotions, such as anxiety and depression, due to a fear of being perceived as vulnerable and/or frail [51] and therefore seeking mental health care depends on the severity of the problem in males [26]. This situation is also reflected in the attitudes of prescribing physicians, who often consider the complaints of men to be more serious in comparison to those of women [28]. Furthermore, contemporary society believes that medications have become indispensible to the normalization of the lives of individuals, as these substances enable the return to routine activities of daily living. The use of medication can also legitimize a disease and affected individual [52].

Antidepressants were the most frequent psychotropic agents used in the population of the city of Campinas, with a higher frequency among the women, which is in agreement with data described in studies conducted in both Brazil [9,32,38] and other countries [3,13]. No significant differences between sexes were found for the other classes of psychotropic drugs. The high consumption of psychotropic drugs, especially antidepressants, may be related to means of diagnosing psychiatric disorders as well as the increase in the indication of this therapeutic class [53]. In the present study, selective serotonin reuptake inhibitors (SSRIs), especially fluoxetine and sertraline, were the most used by both sexes, which is in agreement with data from the previous ISACamp health survey conducted in 2008/2009 [16]. According to the National Therapeutic Chart developed by the Brazilian Health Ministry [54], the management of depression is addressed in several ways, including psychological and pharmacological approaches. For patients with moderate depression, brief psychological treatment in 16 to 20 sessions can be effective, whereas more severe cases should be complemented with pharmacotherapy [54]. The choice of antidepressant to be used is not only based on its effectiveness, but also on other criteria, such as tolerability, safety, toxicity, previous response of the individual or a family manner to a given medication, experience of the prescribing physician regarding the management of a given medication, availability and cost of the medication as well as special situations that require an antidepressant with a lower degree of side effects [54]. A systematic review and meta-analysis of 11 clinical trials showed that SSRIs are as effective as tricyclic antidepressants and have greater tolerability, leading to greater compliance with treatment [55], which may explain the more frequent use of this type of drug.

Some limitations of the present study should be considered when interpreting the results. The cross-sectional design does not enable the determination of cause-and-effect relationships. Therefore, the greater prevalence of psychotropic drug use in individuals without an occupation may stem from the absence or loss of work, which could lead to emotional problems, or, conversely, the occurrence of emotional problems may reduce the possibility of maintaining a job. The quality of the information on medication use should also be considered. However, the care taken to check the package of the medications and medical prescriptions enabled the identification of the pharmacological group in 98.8% of the psychotropic drugs. One should also consider the possibility of recall bias. However, a 15-day recall period is considered adequate and has been widely employed [7,11,38].

The strengths of this study reside in the analysis of data from a representative population of the city of Campinas (state of São Paulo, Brazil) ensured by the complex sampling design. Considering the fact that this was a household survey, the non-response rate was not high and was similar to that found in other studies [56]. This study contributes to the literature by identifying differences between men and women regarding factors associated with the use of psychotropic agents, including differences not observed in other studies, such as the greater consumption among men with white skin, insomnia and a lack of an occupational activity as well as the greater consumption among women with the increase in age and among those with a higher level of schooling.

In conclusion, the present findings confirm the greater use of psychotropic drugs, especially antidepressants, among women and reveal that the pattern of associated factors differs between sexes. It is therefore necessary to understand the peculiarities of each sex that exert an influence on the perception of health problems and the desire to seek care, which, in turn, affect the use of psychotropic agents.

## References

[1] World Bank Group, World Health Organization. Out of the shadows: Making mental health a global development priority [Internet]. 2016. http://www.who.int/mental_health/advocacy/WB_event_2016/en/

[2] Barros M, Lima M, Azevedo R, Medina L, Lopes C, Menezes P, et al Depressão e comportamentos de saúde em adultos brasileiros—PNS 2013. Rev Saúde Pública. 2017;51: Supl 1:8s. 10.1590/S1518-8787.2017051000084 PMC5676399 28591352

[3] Boyd A, Van De Velde S, Vilagut G, De Graaf R, O’Neill S, Florescu S, et al Gender differences in mental disorders and suicidality in Europe: Results from a large cross-sectional population-based study. J Affect Disord. 2015;173: 245–254. 10.1016/j.jad.2014.11.002 25462424 10.1016/j.jad.2014.11.002

[4] Steel Z, Marnane C, Iranpour C, Chey T, Jackson JW, Patel V, et al The global prevalence of common mental disorders: a systematic review and meta-analysis 1980–2013. Int J Epidemiol. 2014;43: 476–493. 10.1093/ije/dyu038 24648481 10.1093/ije/dyu038 PMC3997379

[5] Benjamin J. Sadock Virginia A Sadock PR. Compêndio de Psiquiatria—Ciência do Comportamento e Psiquiatria Clínica. 11th ed Artmed; 2016.

[6] de Loyola Filho AI, Castro-Costa É, Firmo JOA, Peixoto SV. Tendências no uso de antidepressivos entre idosos mais velhos: Projeto Bambuí. Rev Saude Publica. 2014;48: 857–865.26039387

[7] Rodrigues MAP, Facchini LA, Lima MS. Modificações nos padrões de consumo de psicofármacos em localidade do Sul do Brasil. Rev Saude Publica. 2006;40: 107–114. 16410990 10.1590/s0034-89102006000100017

[8] Kantor ED, Rehm CD, Haas JS, Chan AT, Giovannucci EL, HM K. Trends in Prescription Drug Use Among Adults in the United States From 1999–2012. JAMA. 2015;314: 1818–1831. 10.1001/jama.2015.13766 26529160 10.1001/jama.2015.13766 PMC4752169

[9] Quintana MI, Andreoli SB, Moreira FG, Ribeiro WS, Feijo MM, Bressan RA, et al Epidemiology of Psychotropic Drug Use in Rio de Janeiro, Brazil: Gaps in Mental Illness Treatments. PLoS One. 2013;8: 1–7. 10.1371/journal.pone.0062270 PMC3653914 23690934

[10] Quintana MI, Andreoli SB, Peluffo MP, Ribeiro WS, Feijo MM, Bressan RA, et al Psychotropic drug use in São Paulo, Brazil—An epidemiological survey. PLoS One. 2015;10: 1–14. 10.1371/journal.pone.0135059 PMC4529275 26252517

[11] Carrasco-Garrido P, Hernández-Barrera V, Jiménez-Trujillo I, Esteban-Hernández J, Álvaro-Meca A, López-de Andrés A, et al Time Trend in Psychotropic Medication Use in Spain: A Nationwide Population-Based Study. Int J Environ Res Public Health. 2016;13: 1–10. 10.3390/ijerph13121177 PMC5201318 27886138

[12] Blay SL, Fillenbaum GG, Pitta JC, Peluso ET. Factors associated with antidepressant, anxiolytic, and other psychotropic medication use to treat psychiatric symptoms in the city of São Paulo, Brazil. Int Clin Psychopharmacol. 2014;29: 157–165. 10.1097/YIC.0000000000000008 24172159 10.1097/YIC.0000000000000008 PMC3969784

[13] Benson T, O’Neill S, Murphy S, Ferry F, Bunting B. Prevalence and predictors of psychotropic medication use: results from the Northern Ireland Study of Health and Stress. Epidemiol Psychiatr Sci. 2015;24: 542–552. 10.1017/S2045796014000547 25222037 10.1017/S2045796014000547 PMC8367367

[14] Alvarenga JM, de Loyala Filho AI, Araujo Firmo JO, Lima-Costa MF, Uchoa E. A population based study on health conditions associated with the use of benzodiazepines among older adults (The Bambuí Health and Aging Study). Cad Saúde pública. 2009;25: 605–612. 19300849 10.1590/s0102-311x2009000300015

[15] Ribeiro WS, Mari J de J, Quintana MI, Dewey ME, Evans-Lacko S, Vilete LMP, et al The Impact of Epidemic Violence on the Prevalence of Psychiatric Disorders in Sao Paulo and Rio de Janeiro, Brazil. PLoS One. 2013;8: 1–13. 10.1371/journal.pone.0063545 PMC3648507 23667636

[16] do Prado MAMB, Francisco PMSB, de Barros MB A. Uso de medicamentos psicotrópicos em adultos e idosos residentes em Campinas, São Paulo: um estudo transversal de base populacional. Epidemiol e Serviços Saúde. 2017;26: 747–758. 10.5123/S1679-49742017000400007 29211139

[17] Instituto Brasileiro de Geografia e Estatística. Cidades IBGE: Panorama de Campinas [Internet]. 2018. https://cidades.ibge.gov.br/brasil/sp/campinas/panorama

[18] Alves MCGP, Escuder MML, Claro RM, Da Silva NN. Sorteio intradomiciliar em inquéritos de saúde. Rev Saude Publica. 2014;48: 86–93. 10.1590/S0034-8910.2014048004540 24789641 10.1590/S0034-8910.2014048004540 PMC4206113

[19] DEF—Dicionário de Especialidades Farmacêuticas 2015. 43rd ed EPUC; 2015.

[20] World Health Organization. ATC/DDD Index 2016 [Internet]. 2016. https://www.whocc.no/atc_ddd_index/

[21] Gonçalves DM, Stein AT, Kapczinski F. Avaliação de desempenho do Self-Reporting Questionnaire como instrumento de rastreamento psiquiátrico: um estudo comparativo com o Structured Clinical Interview for DSM-IV-TR. Cad Saude Publica. 2008;24: 380–390. 18278285 10.1590/s0102-311x2008000200017

[22] Barros AJD, Hirakata VN. Alternatives for logistic regression in cross-sectional studies: an empirical comparison of models that directly estimate the prevalence ratio. BMC Med Res Methodol. 2003;3: 21 10.1186/1471-2288-3-21 14567763 10.1186/1471-2288-3-21 PMC521200

[23] Fundação SEADE (Fundação Sistema Estadual de Análise de Dados). Perfil dos Municípios Paulistas [Internet]. 2015. http://www.perfil.seade.gov.br/

[24] Dos Santos É G, De Siqueira MM. Prevalência dos transtornos mentais na população adulta brasileira: Uma revisão sistemática de 1997 a 2009. J Bras Psiquiatr. 2010;59: 238–246.

[25] Lima MCP, Menezes PR, Carandina L, Cesar CLG, de A Barros MB, Goldbaum M. Transtornos mentais comuns e uso de psicofármacos: impacto das condições socioeconômicas. Rev Saude Publica. 2008;42: 717–723. 18604365 10.1590/s0034-89102008005000034

[26] Kovess-Masfety V, Boyd A, van de Velde S, de Graaf R, Vilagut G, Haro JM, et al Are there gender differences in service use for mental disorders across countries in the European Union? Results from the EU-World Mental Health survey. J Epidemiol Community Heal. 2014;68: 649–656. 10.1136/jech-2013-202962 24616352

[27] Moreno Luna ME, Clemente Lirola E, Piñero Acín MJ, Martínez Matías MR, Alonso Gómez F, Rodríguez Alcalá FJ. Influencia del género del paciente en el manejo de cuadros ansioso/depresivos. Atención Primaria. 2000;26: 554–558. 11149189 10.1016/S0212-6567(00)78721-X PMC7679645

[28] Gil García E, Romo Avilés N, Poo Ruiz M, Meneses Falcón C, Markez Alonso I, Vega Fuente A. Género y psicofármacos: la opinión de los prescriptores a través de una investigación cualitativa. Atención Primaria. 2005;35: 402–407. 15882496 10.1157/13074791 PMC7668712

[29] Li C, Wilawan K, Samsioe G, Lidfeldt J, Agardh C-D, Nerbrand C. Health profile of middle-aged women: The Women’s Health in the Lund Area (WHILA) study. Eur Soc Hum Reprod Embryol. 2002;17: 1379–1385. 10.1093/humrep/17.5.1379 11980768

[30] Borim FSA, DA Barros MB, Botega NJ. Transtorno mental comum na população idosa: pesquisa de base populacional no Município de Campinas, São Paulo, Brasil. Cad Saude Publica. 2013;29: 1415–1426. 23843008 10.1590/s0102-311x2013000700015

[31] Muñoz Cobos F, Espinosa Almendro JM. Envejecimiento activo y desigualdades de género. Atención Primaria. 2008;40: 305–309. 18588803 10.1157/13123684 PMC7713279

[32] Brunoni AR, Nunes MA, Figueiredo R, Barreto SM, Da Fonseca MDJM, Lotufo PA, et al Patterns of benzodiazepine and antidepressant use among middle-aged adults. the Brazilian longitudinal study of adult health (ELSA-Brasil). J Affect Disord. Elsevier; 2013;151: 71–77. 10.1016/j.jad.2013.05.054 23769607

[33] Paniz VMV, Fassa AG, Facchini LA, Bertoldi AD, Piccini RX, Tomasi E, et al Acesso a medicamentos de uso contínuo em adultos e idosos nas regiões Sul e Nordeste do Brasil. Cad Saude Publica. 2008;24: 267–280. 18278273 10.1590/s0102-311x2008000200005

[34] Olfson M, Marcus SC. National Patterns in Antidepressant Medication Treatment. Arch Gen Psychiatry. 2009;66: 848–856. 10.1001/archgenpsychiatry.2009.81 19652124 10.1001/archgenpsychiatry.2009.81

[35] Han E, Liu GG. Racial Disparities in Prescription Drug Use for Mental Illness among Population in US. J Ment Health Policy Econ. 2005;8: 131–143. 16278501

[36] Gaskin DJ, Briesacher BA, Limcangco R, Brigantti BL. Exploring Racial and Ethnic Disparities in Prescription Drug Spending and Use Among Medicare Beneficiaries. Am J Geriatr Pharmacother. 2006;4: 96–111. 10.1016/j.amjopharm.2006.06.008 16860257 10.1016/j.amjopharm.2006.06.008

[37] Gary FA. Stigma: Barrier to mental health care among ethnic minorities. Issues Ment Health Nurs. 2005;26: 979–999. 10.1080/01612840500280638 16283995 10.1080/01612840500280638

[38] Garcias CMM, Pinheiro RT, Horta BL. Prevalência e fatores associados ao uso de antidepressivos em adultos de área urbana de Pelotas, Rio Grande do Sul, Brasil, em 2006. Cad Saúde Pública. 2008;24: 1565–1571. 18670680 10.1590/s0102-311x2008000700011

[39] Lupi C, Paula A. Mercado de Trabalho: Conjuntura e Análise. IPEA—Inst Pesqui Econômica Apl Ministério do Trab e Emprego. 2012;1.

[40] Marin-Leon L, Oliveira H, Barros M, Dalgalarrondo P, Botega N. Social inequality and common mental disorders. Rev Bras Psiquiatr. 2007;29: 250–253. 17891254 10.1590/s1516-44462006005000060

[41] Batista LE. Masculinidade, raça/cor e saúde. Cien Saude Colet. 2005;10: 71–80.

[42] De Oliveira MM, Daher DV, Da Silva JLL, de A Andrade SSC. A saúde do homem em questão: busca por atendimento na atenção básica de saúde. Cien Saude Colet. 2015;20: 273–278. 10.1590/1413-81232014201.21732013 25650621 10.1590/1413-81232014201.21732013

[43] Pinheiro RS, Viacava F, Travassos C, A dos S Brito. Gênero, morbidade, acesso e utilização de serviços de saúde no Brasil. Cien Saude Colet. 2002;7: 687–707.

[44] Vidal CEL, Yañez FP, Chaves CVS, Yañez CDFP, Michalaros IA, Almeida LAS. Transtornos mentais comuns e uso de psicofármacos em mulheres. Cad Saúde Colet. 2013;21: 457–64.

[45] de Barros MBA, Francisco PMSB, Zanchetta LM, César CLG. Tendências das desigualdades sociais e demográficas na prevalência de doenças crônicas no Brasil, PNAD: 2003–2008. Cien Saude Colet. 2011;16: 3755–3768. 21987319 10.1590/s1413-81232011001000012

[46] Malta DC, Bernal RTI, de Souza M FM, Szwarcwald CL, Lima MG, Barros MB. Social inequalities in the prevalence of self-reported chronic non-communicable diseases in Brazil: national health survey 2013. Int J Equity Health. International Journal for Equity in Health; 2016;15: 153 10.1186/s12939-016-0427-4 27852264 10.1186/s12939-016-0427-4 PMC5112650

[47] Coelho FM, Pinheiro RT, Horta BL, Magalhães PV, Garcias CMM, da Silva CV. Common mental disorders and chronic non-communicable diseases in adults: a population-based study. Cad Saude Publica. 2009;25: 59–67. 19180287 10.1590/s0102-311x2009000100006

[48] Borges TL, Hegadoren KM, Miasso AI. Transtornos mentais comuns e uso de psicofármacos em mulheres atendidas em unidades básicas de saúde em um centro urbano brasileiro. Rev Panam Salud Pública. 2015;38: 195–201. 26757997

[49] Haaramo P, Lallukka T, Lahelma E, Hublin C, Rahkonen O. Insomnia symptoms and subsequent psychotropic medication: a register-linked study with 5-year follow-up. Soc Psychiatry Psychiatr Epidemiol. Springer Berlin Heidelberg; 2014;49: 1993–2002. 10.1007/s00127-014-0862-8 24643300

[50] Sociedade Brasileira do Sono. I Consenso Brasileito de Insônia. Hypnos-Journal Clin Exp Sleep Res. 2003;Supl 2: 9–18.

[51] de Jesus Mari J, Williams P. Misclassification by psychiatric screening questionnaires. J Chronic Dis. 1986;39: 371–378. 3700578 10.1016/0021-9681(86)90123-2

[52] Calmon Reis de Souza Soares J. Abordagens sócio-antropológicas na compreensão do uso do medicamentos. Cad Saúde Colet. 1997;5: 5–12.

[53] Kantorski LP, Jardim VM, Porto Adrize Rutz, S G, Cortes JM, de Oliveira MM. Descrição de oferta e consumo dos psicofármacos em Centros de Atenção Psicossocial na Região Sul brasileira. Rev Esc Enferm USP. 2011;45: 1481–1487. 22241210 10.1590/s0080-62342011000600029

[54] Ministério da Saúde. Formulário Terapêutico Nacional [Internet]. 2010. http://bvsms.saude.gov.br/bvs/publicacoes/formulario_terapeutico_nacional_2010.pdf

[55] MacGillivray S, Arroll B, Hatcher S, Ogston S, Reid I, Sullivan F, et al Efficacy and tolerability of selective serotonin reuptake inhibitors compared with tricyclic antidepressants in depression treated in primary care: systematic review and meta-analysis. BMJ. 2003;326: 1–6. 10.1136/bmj.326.7397.1014 PMC154760 12742924

[56] Mengue SS, Bertoldi AD, Boing AC, Tavares NUL, Pizzol T da SD, Oliveira MA, et al Pesquisa Nacional sobre Acesso, Utilização e Promoção do Uso Racional de Medicamentos (PNAUM): métodos do inquérito domiciliar. Rev Saude Publica. 2016;50: 1–13. 10.1590/S01518-8787.2016050006855ED 26982957

